# Microbiological Load of Edible Insects Found in Belgium

**DOI:** 10.3390/insects8010012

**Published:** 2017-01-13

**Authors:** Rudy Caparros Megido, Sandrine Desmedt, Christophe Blecker, François Béra, Éric Haubruge, Taofic Alabi, Frédéric Francis

**Affiliations:** 1 Functional and Evolutionary Entomology, Gembloux Agro-Bio Tech, University of Liège, Passage des Déportés, 2, Gembloux 5030, Belgium; san.desmedt@hotmail.com (S.D.); e.haubruge@ulg.ac.be (É.H.); t.alabi@ulg.ac.be (T.A.); Frederic.Francis@ulg.ac.be (F.F.); 2 Food Science and Formulation, Gembloux Agro-Bio Tech, University of Liège, Passage des Déportés, 2, Gembloux 5030, Belgium; Christophe.Blecker@ulg.ac.be; 3 Laboratory of Food Process Engineering, Gembloux Agro-Bio Tech, University of Liège, Passage des Déportés, 2, Gembloux 5030, Belgium; F.Bera@ulg.ac.be

**Keywords:** *Tenebrio molitor*, *Acheta domesticus*, *Cirina forda*, Saturniidae, entomophagy

## Abstract

Edible insects are gaining more and more attention as a sustainable source of animal protein for food and feed in the future. In Belgium, some insect products can be found on the market, and consumers are sourcing fresh insects from fishing stores or towards traditional markets to find exotic insects that are illegal and not sanitarily controlled. From this perspective, this study aims to characterize the microbial load of edible insects found in Belgium (i.e., fresh mealworms and house crickets from European farms and smoked termites and caterpillars from a traditional Congolese market) and to evaluate the efficiency of different processing methods (blanching for all species and freeze-drying and sterilization for European species) in reducing microorganism counts. All untreated insect samples had a total aerobic count higher than the limit for fresh minced meat (6.7 log cfu/g). Nevertheless, a species-dependent blanching step has led to a reduction of the total aerobic count under this limit, except for one caterpillar species. Freeze-drying and sterilization treatments on European species were also effective in reducing the total aerobic count. Yeast and mold counts for untreated insects were above the Good Manufacturing Practice limits for raw meat, but all treatments attained a reduction of these microorganisms under this limit. These results confirmed that fresh insects, but also smoked insects from non-European trades, need a cooking step (at least composed of a first blanching step) before consumption. Therefore, blanching timing for each studied insect species is proposed and discussed.

## 1. Introduction

In the context of sustainable development, new methods for production and new food ingredients must be proposed without affecting the quality of food, natural habits, and biodiversity of animal and vegetable species [1,2]. In addition to other propositions, such as algae or cultured meat, insects seem to be a promising solution for the future [1,3,4]. Nevertheless, as for any other foodstuff, particular attention should be paid to microbiological hazards linked with edible insect consumption [5,6]. Effectively, insects are rich in nutrients and moisture and, therefore, provide a favorable environment for microbial growth [4,7]. As an example, Schabel [8] reported some cases of botulism and other foodborne illnesses linked with the consumption of insects stored in poor conditions in Africa. Moreover, the consumption of whole insects, with digestive tracts intact, considerably increases microbiological risks [7]. Consequently, the consumption of crude and whole edible insects should be avoided and a hygienization step by sterilization, pasteurization, blanching (i.e., immersion in hot water in a temperature range from 80–100 °C [9]), or roasting is highly recommended, but processing standards are still needed [2,10].

Few studies have focused on the microbiological hazards presented by insect consumption [9]. The first one was carried out in Africa on a very popular caterpillar: *Gonimbrasia belina* (Westwood 1849) (Lepidoptera: Saturniidae), also known as the mopane worm. Traditionally, the caterpillar undergoes a 24 h fasting period followed by 15 to 30 min of blanching and one to three days of sun drying before being consumed [11,12,13,14,15]. From these processed caterpillars, several species from seven genera of bacteria and five genera of fungi have been identified. Nevertheless, a supplementary boiling step allowed a reduction of the bacterial load. Insect contamination seems to occur during sun-drying, via contact with soil, but also consequently from poor storage conditions [12,14,15]. In their study on reared mealworms (*Tenebrio molitor* L. 1758; Coleoptera: Tenebrionidae), reared house crickets (*Acheta domesticus* (L. 1758); Orthoptera: Gryllidae), and harvested large crickets (*Brachytrupes* sp. Serville 1839; Orthoptera: Gryllidae), Klunder et al. [7] have shown that fresh insects have a high microbiological load, principally composed of Enterobacteriaceae and bacterial spores. This study also demonstrated that a boiling step reduced the number of Enterobacteriaceae and that roasting decreases the bacterial spore load. Another study carried out by Vandeweyer et al. [16] investigated the effects of a short blanching step (10 to 40 s) followed by refrigerated storage or industrial microwave drying on the microbial load of mealworms. They concluded that blanched mealworms can be stored in refrigerated conditions for six days without substantial microbial growth and that blanching (with or without microwave drying) killed vegetative cells, but not bacterial spores. Rumpold et al. [17] studied the surface and volumetric decontamination of mealworms by different techniques (direct and indirect plasma treatment for the surface and high hydrostatic pressure and thermal treatments for the whole insect). An indirect cold plasma technique was effective for surface decontamination and has a potential for live surface sterilization in the rearing process. However, an additional volumetric technique is needed to inactivate the overall microflora, including the gut microbiota. Concerning thermal treatment, a blanching step of 10 to 15 min in a water bath at 90 °C was sufficient to considerably reduce (by three log cycles) the total aerobial count (TAC). These studies emphasized the need for proper processing, packaging, and storage conditions in limiting bacterial loads, especially bacterial spores, in edible insects.

In Belgium, 10 species of edible insects are temporarily tolerated on the market [18]. This tolerance is only valid for whole insects and products based on whole insects produced in the European Union. Nevertheless, as there are no specific criteria for insects sold as human food, the Superior Health Council (SHC) and the Federal Agency for the Safety of the Food Chain (FASFC) of Belgium advised producers to refer to hygiene criteria of minced meat in EU Regulation (EC) No. 1441/2007 [19,20]. Belgian consumers can find a few insects (mealworms, house crickets, and migratory locusts) from the FASFC list in supermarkets, and all these insects are already formulated. Some consumers tend to turn towards unregulated suppliers of insects, such as traditional markets, where other species are sold (Belgium imports at least three tons of mopane worms annually [4]) or pet food shops where fresh mealworms and house crickets are sold. Consequently, this study analyzes the TAC and the yeast and mold count (YMC) of different edible insects species, legalized or not by the FASFC, that can be easily found in Belgium and proposes heat treatment parameters for the reduction of microbial load.

## 2. Material and Methods

### 2.1. Insect Samples

In this section, insects are presented in their initial state of purchase. When a heat treatment was previously applied by the seller, it is described according to the information available. Live mealworms (*Tenebrio molitor*) and house crickets (*Acheta domesticus*) were bought from a local company (Sixlegs SA, Sambreville, Belgium) where they are reared on wheat flour, brewer’s yeast, and wheat bran. As advised by the seller, insects were fasted for 24 h before being killed by freezing, as this procedure is known to reduce the gastrointestinal loads of residual food in the insect gut [21] and, consequently, the insect microbial loads. Effectively, Amadi et al. [22] have shown that the microbial loads of edible insect gut could be higher than the skin microbial loads. As freeze-dried mealworms and house crickets are already found on the Belgian market, they have been bought already processed from a local company (BugsInMugs, Forest, Belgium). This company bought their insects from a supplier, with no information available on the freeze-drying (Fair Insects, Veldhoven, The Netherlands).

Traditionally smoked termites (*Macrotermes* sp. Latreille 1802; Blattodea: Macrotermidae), “yellow Bingula” caterpillars (*unidentified*; Lepidoptera: Saturniidae) and “Mikwati” caterpillars (*Cirina forda* (Westwood, 1892); Lepidoptera: Saturniidae) were bought at a local African market in Matonge (Brussels, Belgium) and were, at least partially, identified following the study of Nsevolo et al. [23]. These three species were collected in the wild in the Democratic Republic of Congo (DRC), but no information was available regarding their processing, shipping, and conservation conditions. The classical process of smoking consists of boiling insects, draining them, and then placing them on metal sieves over a low heat. Smoke is produced by burning wood or sawdust beneath the sieves.

Smoked termites, Mikwati and yellow Bingula caterpillars will be considered as untreated insects in the same way as freshly killed and freeze-dried mealworms and house crickets.

### 2.2. Heating Treatment

Firstly, a blanching time was determined for each previously described, except for freeze-dried insects, which were considered as “ready-to-eat”. For this purpose, each insect was weighed on an analytical balance (Sartorius AX224, Goettingen, Germany) and had its diameter measured using a 10-cm sliding caliper (VWR, Radnor, PA, USA). Due to their bigger size, yellow Bingula caterpillars, Mikwati caterpillars and freshly killed house crickets could be coupled with a temperature sensor (1.2 mm, Ellab, Hillerød, Denmark) connected to a temperature recording system (TM9608, Ellab, Hillerød, Denmark) to determine their blanching time. Once coupled, insects were immersed in a 99 °C (±0.5 °C) and thermostatically controlled water bath (Polystat 71, Huber, Offenburg, Germany) until insects reached a 100 °C core temperature. Freshly killed mealworms and smoked termites, which were too small for the temperature sensor, were heated for 1 min in a 99 °C (± 0.5°C) water bath. All insects were directly cooled in cold water after blanching. Secondly, the species-dependent blanching time previously determined was applied to nine replicates for each insect species for further microbiological analyses.

Freshly killed mealworms and house crickets were also sterilized in cans to evaluate the impact of a sterilization treatment. Batches of 60 g of each insect were placed in tin cans filled with 60 mL of brine solution (NaCl 5% in water). Cans were then crimped and autoclaved for 16 min at 120 °C. One can was equipped with a thermocouple in order to monitor the temperature inside the can during the temperature program.

### 2.3. Microbiological Analyses

Insect samples (30 g for each species under each treatment) were crushed in a universal blender (M20; IKA, Stauffen, Germany). The blender’s grinding chamber was autoclaved before utilization. Ten grams of ground material (*n* = 3) was suspended in 90 mL sterile isotonic quarter-strength Ringer solution (ref. BR001; Biokar, Beauvais, France) in a Stomacher^®^ plastic bag (Seward, Worthing, United-Kingdom) and homogenized in a paddle blender (80 Biomaster, Seward, Worthing, United-Kingdom) for 1 min. The suspensions were serially diluted 10-fold (up to 10^−8^) in isotonic quarter-strength Ringer solution. One milliliter of suspension was plated on each of two media. Total numbers of aerobic mesophilic microorganisms were determined on plate count agar (BioRad, Hercules, CA, USA) after incubation at 30 °C for 72 h. Yeasts and molds were determined on oxytetracycline-glucose-yeast extract agar (Biokar, Beauvais, France) after incubation at 25 ± 1 °C for 120 h.

### 2.4. Hygiene Criteria

The Commission of the European Communities (TCEC) published limit criteria on TAC (m (low limit) = 5.70 cfu/g and M (high limit) = 6.70 log cfu/g for five sample units tested from the food batch, with at least two sample units giving values between m and M; [24]). Values above M are unacceptable in the terms of the sampling plan and would cause rejection of the batch. Our results will be compared with the criteria mentioned in EU Regulation (EC) No. 1441/2007, even if we are only investigating three sample units instead of the recommended five. For YMC, the guidelines of Good Manufacturing Practices for raw meat, described by Stannard [25], advising a YMC < 4 were followed.

### 2.5. Statistical Analysis

One-way ANOVAs with a 95% confidence interval were used to evaluate the influence of different heat treatments on the TAC and YMC of the insect species studied and were performed using Minitab^®^ (ver. 16.0 for Windows^®^; Minitab, Inc., State College, PA, USA).

## 3. Results

Yellow Bingula caterpillars (average weight = 0.77 g ± 0.19 g), Mikwati caterpillars (2.68 g ± 0.96 g), and house crickets (0.26 g ± 0.04 g) needed, respectively, 4.17 min ± 0.69 min, 4.13 min ± 1.16 min, and 3.16 min ± 1.04 min of blanching to reach a core temperature of 100 °C. Following these results, it was decided that blanching treatments of 4 and 5 min would be applied to the house crickets and the two caterpillar species, respectively. As explained in the Material and Methods, mealworms (0.07 g ± 0.01 g) and termites (0.08 g ± 0.01 g) are only 2 mm in diameter and were incompatible with the thermocouple sensor. Considering the limited average weight of these two insect species, a heat treatment of blanching for 1 min was applied. Table 1 shows that all freshly killed and smoked insect samples had a total aerobic count (TAC) higher than the high limit M for fresh minced meat (6.70 log cfu/g; TCEC [24]), but also that all blanching treatments reduced TAC under this limit, except for the Mikwati caterpillars.

The application of freeze-drying and sterilization to mealworms and house crickets also showed a reduction of TAC under the fresh minced meat limit. Nevertheless, sterilization seems slightly more effective than blanching and freeze-drying. Concerning yeast and mold counts (YMC), all treatments prevented yeast and mold development in each of the insects, for at least 5 days storage at 25 ± 1 °C.

## 4. Discussion

Freshly killed and smoked insects analyzed in this study showed a total aerobic count (TAC) and yeast and molds count (YMC) superior (or very close) to the advised limit for that of fresh minced meat (log cfu/g; TAC < 6,70 and YMC < 4) [24,25]. The TAC and YMC of fresh mealworms (respectively, 8.58 ± 0.07 and 4.70 ± 0.46) were consistent with other studies, showing a TAC level from 7.7 to 8.3 log cfu/g and a YMC level from 3.5 to 5.7 log cfu/g [7,17,20]. The TAC of fresh house crickets (7.97 ± 0.10) was also consistent with the results of Klunder et al. [7], showing a TAC level of 7.2 log cfu/g. This high contamination level could be explained by the presence of microorganisms in the insect gut, despite a fasting step [4,7], and by rearing conditions that could affect the presence and growth of potential spoilage bacteria and food pathogens [19]. No relevant literature was found on the microbiological quality of the three traditionally smoked species. Braide et al. [26] studied the effect of biodeterioration on the microbiology of fried termites (*Macrotermes bellicosus* (Smeathman, 1781); Blattodea: Macrotermidae). Unfortunately, only data on samples following one week of deterioration at ambient temperatures have been published. Not surprisingly, microbiological analyses on these samples showed high levels of microorganisms involved in food deterioration and spoilage [26]. Igbabul et al. [27] studied the microbial quality of dried larva of Mikwati caterpillar bought from Nigerian markets, and found a low microbial load (maximum of 3.6 log cfu/g) in their sample. This could be explained by sun drying of the dried larvae, assessed by Igbabul et al. [27], before microbiological analyses. On the other hand, Braide et al. [28] showed high microbial loads (7.65 log cfu/g) in emperor moth larvae (*Bunaea alcinoe* (Stoll, 1780); Lepidoptera: Saturniidae), freshly harvested on leaves and traditionally smoked and sun dried before microbiological analyses. The high levels of contamination of smoked insects found in this study and by Braide et al. [28] could be explained by an unhygienic method of processing, packaging, storage, or marketing in the harvesting country that could lead to recontamination. Effectively, for example, relatives bring most of the exotic insects found on Belgian traditional markets back after a trip in their home country without following any sanitary regulations, leading to product spoilage. Additional processing steps, such as a vinegar treatment or the use of a modified atmospheric packaging system, could probably improve the microbiological quality of these products, but still needs to be studied [28].

Despite the high TAC and YMC in untreated, freshly killed or smoked insects, a blanching step has been shown to be sufficient to decrease the microbial and fungal loads under fresh minced meat limits in all samples except for Mikwati caterpillar TAC. These results are consistent with the studies of Klunder et al. [7] and Vandeweyer et al. [16], demonstrating the effectiveness of blanching treatment (respectively, 10 min for *Tenebrio molitor* or 5 min for *Acheta domesticus* and 20 s for *Tenebrio molitor*) in eliminating Enterobacteriaceae, but not spore-forming bacteria. To reduce the load of bacterial spores, an additional processing step is clearly needed or the product will have a very short shelf life. Vandeweyer et al. [16] have shown that a microwave-drying step after blanching could represent a pasteurization treatment, helping to kill vegetative cells with little or no effect on spores, as they only dehydrated the product. In our study, sterilization was the most efficient treatment for reducing TAC and YMC in mealworms and house cricket; however, following the results of Vandeweyer et al. [16], a precise characterization of the remaining microbiota is clearly essential to ensure that the product is harmless while sterilization is known to kill bacterial spores.

Reduced TAC and YMC in freeze-dried insects show that freeze-drying could be considered as an effective processing technique. Nevertheless, this technique is known to mostly inactivate microorganisms rather than kill them, and, when rehydrated, many could return to a vegetative stage that could be harmful [29]. Consequently, some authors recommend applying a heat treatment to freeze-dried insects before consuming them [29], but no data are available on the specifications of this treatment. The freeze-drying conditions of the insects used for this study were unfortunately not available. Nevertheless, 72 h of freeze-drying (at −50 °C) of mealworms and house crickets was shown to be ineffective in TAC reduction (personal data in [2]).

Finally, this study is the first to show that processed insects from illegal markets are highly contaminated with microorganisms and, as with fresh insects, require a processing step before consumption. As these insects are easily available in Belgium (three tons of mopane worms are imported to Belgium each year), our results show that there is a need for a precise legislative framework on the trade of wild harvested edible insects and standards for their processing, packaging, labeling, and storage, which could result in the establishment of an international certification. As most of the edible insects found in Belgium seem to need a thermal treatment before consumption, Western consumers need information on proper ways to consume edible insects. Effectively, the SHC and FASFC, in Belgium, published consumption advice in their report on edible insects (e.g., legs and wings from Orthoptera are inedible or the consumption of fresh insects or insects from feed producers must be avoided) that are unknown to the general public [20]. Moreover, current legislation on edible insects must be quickly reinforced and be more focused on spore-forming bacteria.

## 5. Conclusions

This study confirms that a species-specific processing step with an anti-microbial effect is required in order to avoid or minimize risks involved with the consumption of edible insects from European farms, but also for edible insects from non-European traditional trades. Specific attention must be paid to non-European species that are supposed to be ready-to-eat, as they are already processed but need a supplementary processing step. The establishment of international certification for edible insects could be intended as a way to ensure safer products for consumers. The next step in the development of the edible insect sector seems to be the selection of proper microbial, inactivation, packaging, and storage technologies, including technologies that specifically address spore-forming bacteria.

## Figures and Tables

**Table 1:** Effect of different treatments on the total aerobic and yeast and molds counts (log cfu/g) of different edible insect species found in Belgium (*n* = 3; DRC = from Democratic Republic of Congo; EF = from European farms; statistical analyses = one way ANOVAs for each species with 95% confidence interval; *F* = F statistic; *p*: significance level; different letters indicate significant differences between treatments). Freeze-dried and smoked insects were bought already processed.

Insect Sample	Treatment	Total Aerobic Count	Statistical Analyses	Yeasts and Molds	Statistical Analyses
Freshly killed mealworm (EF)	no treatment	8.58 ± 0.07 ^a^	*F*_3,7_ = 1538.58; ***p* < 0.001**	4.70 ± 0.46 ^a^	*F*_3,7_ = 23.18; ***p* = 0.005**
	1 min blanching	4.64 ± 0.07 ^b^		< 1.0 ± 0.00 ^b^	
	sterilized	3.34 ± 0.06 ^c^		< 1.0 ± 0.00 ^b^	
Freeze-dried mealworm (EF)	no treatment	4.47 ± 0.12 ^b^		< 1.0 ± 0.00 ^b^	
Freshly killed house cricket (EF)	no treatment	7.97 ± 0.10 ^a^	*F*_3,7_ = 904.32; ***p* < 0.001**	4.80 ± 0.29 ^a^	*F*_3,7_ = 69.31; ***p* = 0.001**
	4 min blanching	4.39 ± 0.01 ^b^		< 1.0 ± 0.00 ^b^	
	Sterilized	3.74 ± 0.14 ^c^		< 1.0 ± 0.00 ^b^	
Freeze-dried house cricket (EF)	No treatment	4.05 ± 0.07 ^bc^		< 1.0 ± 0.00 ^b^	
Smoked termite (DRC)	No treatment	7.48 ± 0.04 ^a^	*F*_1,3_ = 365.95; ***p* = 0.003**	5.62 ± 0.32 ^a^	*F*_1,3_ = 632.06; ***p* = 0.002**
	1 min blanching	4.45 ± 0.22 ^b^		< 1.0 ± 0.00 ^b^	
Smoked yellow Bingula caterpillar (DRC)	no treatment	6.84 ± 0.10 ^a^	*F*_1,3_ = 285.33; ***p* = 0.003**	3.58 ± 0.18 ^a^	*F*_1,3_ = 836.18; ***p* = 0.001**
	5 min blanching	3.64 ± 0.25 ^b^		< 1.0 ± 0.00 ^b^	
Smoked Mikwati caterpillar (DRC)	no treatment	7.63 ± 0.01 ^a^	*F*_1,3_ = 1.07; *p* = 0.410	5.67 ± 0.10 ^a^	*F*_1,3_ = 6726.25; ***p* < 0.001**
	5 min blanching	7.32 ± 0.43 ^a^		< 1.0 ± 0.00 ^b^	

Bold values indicated significant results.
